# Colonization of Dental Unit Waterlines by *Helicobacter pylori*: Risk of Exposure in Dental Practices

**DOI:** 10.3390/ijerph16162981

**Published:** 2019-08-19

**Authors:** Monica Giacomuzzi, Carla M. Zotti, Savina Ditommaso

**Affiliations:** Department of Public Health and Pediatrics, University of Turin, Piazza Polonia 94, 10126 Turin, Italy

**Keywords:** dentistry, dental unit, infection control, cross-infection contamination, *Helicobacter pylori*, streptococci

## Abstract

Dental unit waterlines (DUWLs) can be considered one of the possible routes of *H. pylori* transmission, although its presence in DUWLs has not yet been investigated thoroughly. The present study aimed to discover the prevalence of *H. pylori* and oral streptococci (*S. oralis* and *S. mutans*) in DUWLs to evaluate the risk of exposure to human pathogens in dental practices. We collected the output water from 60 dental chair units (DCUs) in 26 private dentistry settings in Turin, searching for *H. pylori* and oral streptococci (OS) DNA, with a polymerase chain reaction (PCR) technique. At the same time, dentists completed a questionnaire about their DCUs, their main activities, the presence of anti-retraction devices, their attitudes about disinfection, etc. No dental chair unit tested was contaminated with *H. pylori* or *S. mutans*; only one dental chair was contaminated with *S. oralis* (1.7%). Considering the results, we can state that: (i) the lack of *H. pylori* DNA in water samples analyzed, suggests that municipal water is presumably treated with a sufficient chlorine level to inactivate DNA over time; (ii) the aspiration of oral fluids is limited by anti-retraction valves fitted distally to hand pieces; (iii) propidium monoazide qPCR (PMA-qPCR) could be a good technique to investigate and monitor potential environmental sources of infections such as DUWLs.

## 1. Introduction

Thirty-four years after the first confirmed cultivation and identification of *Helicobacter pylori* by the Australian physicians Robin Warren and Barry Marshall [1], the scientific community is still attempting to find out the transmission dynamics of this gastric pathogen.

Many pathways of transmission have been hypothesized, mainly based on epidemiological and microbiological data: five of the suggested routes are characteristic of direct person-to-person transmission (breastfeeding, iatrogenic, oral-oral, gastro-oral, and fecal-oral pathways) [2,3,4,5] that, based on the intrafamilial clustering observed, appears to be the main route. Another four possible transmission routes are waterborne, zoonotic, milk ingestion-based, and raw vegetable-based [6,7,8], each of which requires a contaminated intermediate environmental reservoir; for the above examples, these reservoirs are water, animals, and vegetables.

The prevalence of *H. pylori* infection is extremely variable across different geographical areas, even though the precise numbers change between studies. *H. pylori* infection is common in non- industrialized countries, where the prevalence of infection is assumed to be more than 80% in middle-aged people, whereas in developed countries it is estimated that only 20% to 50% of the population carries *H. pylori* [9] In general, lower socioeconomic status (i.e., level of hygiene, density of living, and educational opportunities) correlates with an increased prevalence of *H. pylori* infection. This higher prevalence of infection in non-industrialized countries suggests that hygiene and environmental factors may play important roles in transmission, with water being a plausible origin of *H. pylori* infection either via ingestion of contaminated water or by contact with water-related items [10].

The validity of the theory of contaminated water as a pathway of transmission is well supported because water biofilms are believed to protect microorganisms [11], and moreover because this theory has been confirmed by epidemiological studies showing the association between *H. pylori* prevalence and water-related sources [12,13], as well as by studies that isolated *H. pylori* from water sources [14,15].

Dental unit waterlines (DUWLs) become contaminated with micro-organisms from two sources. First, municipal water, if used in DUWL (some chairs use a reservoir bottle) and second from the oral cavities of patients by aspiration of biological fluid during therapy (when the air turbine handpieces stop rotating). If dental patients are pathogen carriers, microorganisms can be transmitted by direct contact or via dissemination by aerosol sprays created by dental handpieces (high-speed drills, scalers, air or water syringes) to subsequent patients. Nowadays, a large numbers of dental water systems are fitted with anti-retraction valves to stop suck-back of oral contaminants and/or give a short ‘terminal flush’ of water to remove any suck-back material [16].

The environmental and human bacteria adhere to the walls of the dental unit and establish a biofilm.

Dental unit waterlines (DUWLs) can be considered one of the possible routes of *H. pylori* transmission, but this organism’s presence in DUWLs has not yet been investigated thoroughly by scientific research.

After performing a review of the scientific literature, we found that only one Iranian study exists to date on this topic [17]; this study reports that this pathogen was found in 11.4% of DUWLs; the only additional data were reported by A. Sajadi [18] at the conference of the International Association for Dental Research (IADR) in San Diego and these data showed that *H. pylori* was found in 23 biofilm samples obtained from 30 DUWLs.

*H. pylori* contamination in DUWLs can be derived from the oral fluid of the person undergoing dental treatment and from the municipal drinking water supply.

The prevalence of *H. pylori* in the oral cavity varies from 0% to 65% [19,20,21,22]. The conflicting results in published works may be caused by differences in geographic area (the acquisition of *H. pylori* infection was correlated with socioeconomic status) [23], sample collection, diagnostic techniques, or oral contamination from gastric juice as a result of gastro-esophageal reflux at the time of sampling.

The bacteria can persist in water due to its ability to adapt to harsh conditions, during which it becomes virtually metabolically inactive, entering a viable but nonculturable (VBNC) state. Under these conditions, the organism maintains its metabolic activity and pathogenicity, and it may revert to active regrowth conditions through a conversion from a spiral into a coccoid form [11,24,25]. Entrance of *H. pylori* into the VBNC state allows the bacterium to survive in water, although it rapidly loses its cultivability; as a consequence, attempts to culture *H. pylori* cells from environmental water samples have largely been unsuccessful.

Traditional culturing methods are extremely limited, mainly due to the absence of an optimal selective culture medium: some culture media are too nutrient rich and cause nutritional shock, which can hamper the bacterial growth in culture plates, or the nutrients support overgrowth by competing microorganisms. Therefore, most methods used to detect *H. pylori* in environmental samples are based on culture-independent molecular techniques such as polymerase chain reaction (PCR), quantitative real-time polymerase chain reaction (qPCR) or fluorescent in situ hybridization (FISH).

Despite the high sensitivity and specificity of molecular methods to identify *H. pylori* in the environment, it is important to realize that they suffer from an important limitation: these techniques cannot distinguish between viable and dead bacteria [15]. Different methods have been developed to detect and differentiate between viable cells by PCR or qPCR, such as the use of the DNA- intercalating fluorophore propidium monoazide (PMA). PMA is a high affinity, photoreactive dye that preferentially binds to dsDNA. Intact cell membranes are impermeable to PMA, and therefore, it only penetrates the damaged membranes of dead cells to intercalate into the DNA; this intercalation blocks amplification during PCR [26,27].

This study was conducted to evaluate the risk of *H. pylori* exposure in dental practices. To this end, the occurrence of this bacteria in samples of output water from dental unit water lines (DUWLs) was tested using a quantitative PCR technique (qPCR), combined with pretreatment of the samples with PMA. The samples were collected in 26 private dentistry settings in Turin. In addition, to evaluate the success rate of anti-retraction devices, we used qPCR to search for the presence of oral streptococci (OS), specifically *Streptococcus mutans* and *Streptococcus oralis*, in samples from DUWLs, as these bacteria can serve as biological marker of water contamination by oral fluids.

## 2. Materials and Methods

In 2015, 86 water samples taken from DUWLs and sink faucets (tap water) of 26 private dentistry settings underwent microbiological examination (Supplementary Materials S1).

The sample taken from the tap was used to check the quality of the water supplied to the building where the dental office was situated.

### 2.1. Sampling Water from DUWLs

Water samples (1 L) were obtained in the morning for every unit from two different operative sites: the air-water syringes and turbines, and then were mixed together. Each sample was aseptically collected in a sterile plastic bottle containing sodium thiosulphate at a final concentration of 0.01% w/v to neutralize the residual chlorine in water.

### 2.2. Sampling Water from the Tap

Before water collection, the taps were sanitized using these four steps: (1) the water-flow regulator was removed; (2) the inside of the tap was disinfected using sodium hypochlorite (10% w/v) for 2–3 min; (3) the external part of the faucet was flamed using a Bunsen burner; (4) the water was allowed to flow for 5 min. At the end of this procedure 1 L of water was collected in sterile plastic bottles with sodium thiosulphate at a final concentration of 0.01% w/v.

### 2.3. DNA Extraction and PMA Treatment

Each 1-L water sample was transported to the laboratory and processed within 2 h. Then it was filtered through a 0.45 μm pore size polycarbonate filter (Millipore, Billerica, MA, USA) according to the manufacturer’s instructions (Aquadien, Bio-Rad, Marnes-la-Coquette, France). The filter was overlaid with 500 μL of PMA (50 μM) in a 90-mm Petri dish and was then incubated in the dark for 10 min, after which it was placed on ice and exposed to a 500 W light for 10 min at a distance of 20 cm from the light source. After irradiation, the filter was placed in 2 mL of lysis solution for DNA extraction. To eliminate the bacterial resuspension step, which could cause the loss of some bacteria, the DNA was extracted directly from the bacteria on the filters. The conditions for this process were optimized in our previous study [27].

### 2.4. Quantification of Viable *H. pylori* and Oral Streptococci Using PMA-qPCR

The extracted genomic DNA was analyzed for the presence of amplifiable sequences using three qPCR commercial kits for the detection of *H. pylori*, *S. oralis*, and *S. mutans* according to the manufacturer’s instructions (Primerdesign^TM^ Ltd.) (Camberley, UK). The kit for *H. pylori* detection contains the reagents to amplify and quantify a 100-bp fragment of the gene segment coding for the beta subunit of RNA polymerase (rpoB), the kit for *S. oralis* amplifies a segment of the glucosyltransferase (gtfR) gene, and the kit for *S. mutans* detection amplifies a segment of the glucosyltransferase-I gene (gtfb), previously identified as a highly specific marker for *S. mutans*.

All the three kits provide amplification solutions containing Taq DNA polymerase, an internal control, primers, fluorescent Taqman probes, a negative control, and a positive control, the latter of which are used as standards for quantification. Under optimal PCR conditions, Primer Design detection kits can detect less than 100 copies of target template. The internal control allows the detection of any possible factors that might inhibit the amplification reaction and is amplified in the same reaction mixture; however, different primers and a probe labeled with a different fluorophore were used.

The qPCR data were analyzed using Opticon Monitor Analysis Software version 3.4 (Bio-Rad, Hercules, CA, USA).

### 2.5. Risk Assessment Questionnaire and Survey of General Dental Practice Attitudes

Dentists working at general dental surgeries using dental units in their daily clinical practice were asked to voluntarily answer 15 questions included in an anonymous questionnaire. The survey questionnaire (Supplementary Materials S2) was developed for this study and comprises a set of general questions about the dental chair units (DCUs): the type of water system, the age, the manufacturer, the model. The dentists were also questioned about their main activities (oral surgery, dental hygiene, or conservative dentistry), the presence of anti-retraction devices, the predisposition for continued or discontinued disinfection, about their attitudes about cleaning and disinfecting the DCUs for risk containment, and about the use of microbiological testing of DUWL water in their dental settings.

## 3. Results

### 3.1. Detection of *H. pylori* and Oral Streptococci by PMA-qPCR

A total of 26 water samples from sinks (tap water) and 60 water samples from DUWLs were collected and analyzed using the PMA-qPCR methods designed to detect *H. pylori* and oral streptococci.

No dental chair unit tested was contaminated with *H. pylori* or *S. mutans*. The amplification of internal control DNA assays for these samples demonstrated that they did not hold important levels of inhibitory or interfering compounds.

Only one dental chair was contaminated with *S. oralis* (1.7%). The genomic unit (GU) values for this dental chair samples determined using the PMA-qPCR method was 211 GU/L, showed that the concentration of *S. oralis* was low. This dental chair was equipped with anti-retraction valves.

The genomes of the three target microorganisms were not amplified from any water sample taken from the sinks of the offices (control).

### 3.2. Questionnaire Answers

All DCUs were directly connected to the municipal water supply, and 5% were equipped with a filter in the water input, while 13% were equipped with a water softening system.

The average age was 10 years (ranging from 1 to 30 years). In regard to dental treatment, the main activity was conservative dentistry (61%), followed by dental hygiene (25%) and oral surgery (14%). The most common DCUs manufacturer was Castellini (*n* = 12), followed by Eurodent (*n* = 11) and Sternweber (*n* = 7), while other manufacturers were represented with a few units each. Moreover, 75% of the DCUs were equipped with anti-retraction valves.

In regard to the presence of disinfection systems, 74% of the DCUs were equipped with disinfection systems and were continuously or intermittently sanitized, and the chemical product that was mostly commonly used (50%) was hydrogen peroxide 3%.

None of the respondents usually perform microbiological testing of the DUWLs output water in their dental practice.

## 4. Discussion

The microorganisms that contaminate DUWLs can originate from the water supply and from biological fluids from patients during dental therapy. The environmental bacteria dispersed in the inlet water settle on the inner surface of the DUWLs and create a biofilm. Human pathogens from saliva and other oral fluids, as well as blood in cases of invasive therapy can enter DUWLs through aspiration of biological fluids due to the temporary negative pressure generated when the drill ends up rotating.

If we presume that OS are a surrogate marker of contamination by oral secretions, because they exclusively colonize the upper aerodigestive tract of mammals and they do not become active members of biofilm, then their detection in DUWLs suggests the occurance of biological fluid aspiration from dental patients. As a result, the identification of OS in DUWLs would sustain the theory that the risk of blood or air-borne infections for successive patients is greater than zero [28,29,30]. Also the colonization of the DUWLs by *H. pylori* may occur via contamination of the inlet water or from human oral fluids. This colonization can be limited by waterline disinfection and by anti-retraction valves fitted to the hand pieces.

The present study aimed to discover the prevalence of *H. pylori* and oral streptococci (*S. oralis* and *S. mutans*) to evaluate the risk of exposure to human pathogens in dental practices.

Drinking water samples collected from sink faucets (tap water) were examined as a control, and in none of these samples did we identify detectable *H. pylori* or OS DNA.

This result was not unpredictable since drinkable water supplied by aqueducts undergoes preliminary potabilization which, in Italy, is mainly accomplished by chlorine addition. Chlorine disinfection of drinking water remains one of the primary means of preventing the spread of waterborne disease. After potabilization, heterotrophic bacterial counts of output water must comply with the threshold values established by the European Council Directive 98/83/EC [31] (20 CFU/mL at 36 ℃ and 100 CFU/mL at 22 ℃) and the accepted free chlorine concentration of 0.2 mg/L. As was demonstrated by Johnson et al. [32], *H. pylori* is sensitive to chlorine and readily inactivated by free chlorine (0.5 mg of free chlorine per liter) and should therefore be controlled by disinfection practices normally employed in the treatment of drinking water. Isolation of viable *H. pylori* from water has been reported in developing countries, with less optimal water hygiene, suggesting that bacterial isolation is more likely to be successful when the microbial burden is relatively high. Examples include studies in Pakistan, Iraq, and Iran [33].

Dental unit water samples collected from 60 DUWLs were tested in this research, but the *H. pylori* DNA was not detected. The prevalence rate of OS (*S. oralis*) contamination was 1.7%.

The absence of *H. pylori* and the low prevalence of OS can be attributed both to the effective disinfection of municipal drinking water, to the good functionality of the valves, and to correct maintenance procedure of the dental unit.

The low prevalence of OS DNA could also be related to the kind of dental treatment carried out, that is, the invasiveness and length of the treatment and the kind of hand device used.

The little amount of information on the frequency of recovery of OS by culture from DUWLs demonstrated prevalence values ranging from 0% to 34% [29,34,35,36]; however, this discrepancy could be due to the use of diverse microbiological procedures in the different studies and/or to misclassification of other microorganisms as oral streptococci.

Likewise, in previously published reports the studies that reported *H. pylori* DNA in 11.4% and 23% of DUWLs [17,18], involved analyses performed without propidium monoazide (PMA) pretreatment, thus without distinguishing between viable and dead bacteria and potentially overestimating the *H. pylori* amount. PMA is a membrane-impermeant dye that only enters bacterial cells with compromised membranes, where it is then cross-linked to the DNA, thus inhibiting PCR amplification. Our method included a PMA treatment in order to eliminate the background of dead cells.

## 5. Conclusions

The results of the present investigation show that OS were detected in water from only one dental unit and never in water from tap water, suggesting that OS are not present in the incoming water but are aspirated into DUWs during dental therapy. However, the low concentration of OS suggests that only a small volume of oral fluids is aspired and the risk of exposure of successive patients to the biological fluids is very low. Moreover, the lack of *H. pylori* DNA in the water samples analyzed, suggest that (i) municipal water is presumably treated with sufficient chlorine levels to inactivate the DNA over time; (ii) the aspiration of oral fluids is limited by anti-retraction valves fitted distally to hand pieces.

In conclusion we would like to say that PMA-qPCR could be a good technique to determine *H. pylori* viability in environmental samples and to investigate and monitor potential environmental sources of infections such as DUWLs.

## Supplementary Materials

The following are available online at https://www.mdpi.com/1660-4601/16/16/2981/s1, S1: dental chair unit monitoring, S2: Translated questionnaire, risk assessment questionnaire and survey of general dental practice attitude.
