# Supplementary material for: Colonization of Dental Unit Waterlines by Helicobacter pylori: Risk of Exposure in Dental Practices
Source: Int J Environ Res Public Health. 2019 Aug 19;16(16):2981. doi: 10.3390/ijerph16162981 (PMC6727081; doi:10.3390/ijerph16162981)
Supplement: Supplementary file 1 [file ijerph-16-02981-s001.zip › ijerph-558142-supplementary-S2.pdf]

Record n° é é é é é é é é é é é é

Dental unit (brand / model) é é é é é é é é é é é installation date é .é é é é é é é é ...

Main activity (i.e. dental hygiene, conservative, surgery,etc) ...é .é é é é é é é é é é ..é é é ...é

No. of working days per week é é é é ..é é .. No. of patients per day é é é é é é é é ...

The dental unit clinical irrigator for the working handpieces and for the rinsing cup is :

☐ **potable water derived from the building's cold water supply**

☐ **water or other liquid supplied by an independent water reservoir**

Is there a system for the softening of the incoming water ? yes☐ no☐

Is the dental unit equipped with a filter for the incoming water? yes☐ no☐

Is sterile water used as a coolant/irrigator when performing surgical procedure? yes☐ no☐

Is there a physical air gap separating DUWLs from the municipal water supply to prevent the back-siphonage of clinical material? yes☐ no☐

Is the dental unit equipped with anti-retraction devices toward the working handpieces (i.e. the high-speed handpiece, air/water syringe , ultrasonic scaler etc)? yes☐ no☐

Is the dental unit equipped with a disinfection system? yes☐ no☐

Is the dental unit disinfected? yes☐ no☐

If yes, with a continue or discontinue system? é

In case of continue disinfection which product is used? é é é é é é é é é ..é é é .é é é é é

at which concentration? é .é .é é é .

In case of discontinue disinfection which product is used? é é é é é é é é é é é é .é é

with which frequency? é é é é é é é é é é é at which concentration? é é .é é é .é é .

and contact time? é é é é é é é é ...é é ...

Do you disinfected the tubing system that drains liquids off the unit? yes☐ no☐

If yes, with which frequency? é .

Do you monitor the dental water quality to assess its microbiological composition? yes☐ no☐

If yes, with which frequency? é .

Is the dental unit subjected to routine maintenance by the manufacturer? yes☐ no☐

If yes, with which frequency? é .
