# Supplementary figures and images for: Colonization of Dental Unit Waterlines by Helicobacter pylori: Risk of Exposure in Dental Practices
Source: Int J Environ Res Public Health. 2019 Aug 19;16(16):2981. doi: 10.3390/ijerph16162981 (PMC6727081; doi:10.3390/ijerph16162981)

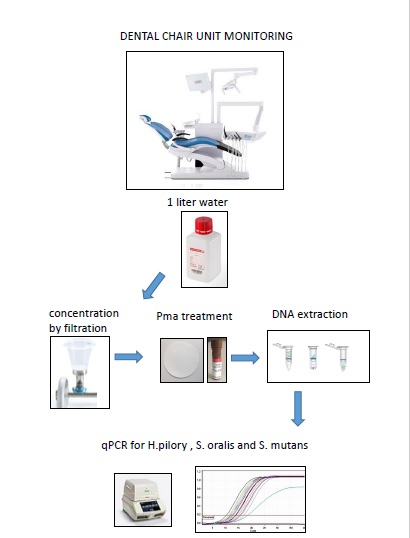

Supplement: Supplementary file 1 [file ijerph-16-02981-s001.zip › ijerph-558142-S1.jpg]
